# Does a Dual-Mobility Cup Offer Better Stability than Conventional Bearings in Hip Arthroplasty Following Femoral Neck Fracture?

**DOI:** 10.3390/jcm14165613

**Published:** 2025-08-08

**Authors:** Itay Ron, Itay Ashkenazi, Nimrod Snir, Yaniv Warschawski, Aviram Gold

**Affiliations:** 1 The Ruth and Bruce Rappaport Faculty of Medicine, Technion—Israel Institute of Technology, Haifa 3525433, Israel; itayron2000@gmail.com; 2 Division of Orthopedics, Tel Aviv Sourasky Medical Center, 6 Weizman Street, Tel-Aviv 6423906, Israelyanivarsh@gmail.com (Y.W.)

**Keywords:** arthroplasty, hip, hip fractures, femoral neck fractures, prostheses, implants, dual mobility bearings

## Abstract

**Introduction:** Instability following total hip arthroplasty (THA) remains a challenging complication. Dual-mobility (DM) hip components are aimed at improving joint stability by increasing the head-neck ratio and jump distance. However, data regarding the efficacy of these implants in the trauma setting are scarce. This study aimed to compare the dislocation rates of DM bearings with conventional THA in patients undergoing primary THA for the treatment of hip fractures. **Methods:** We retrospectively reviewed all patients who underwent THA for hip fractures between the years 2010–2022 and had a minimum follow-up of two years. Patient demographics and radiographic parameters, including cup version, leg length discrepancy (LLD) and femoral horizontal offset, were compared between patients who received DM bearings and patients who received conventional THA. Dislocation and revision surgery rates were also compared between the groups. **Results:** The study included 570 patients who met inclusion criteria, of which 82 patients were in the DM bearings group and 488 patients were in the conventional THA group. Baseline demographics and comorbidity profiles were comparable between the groups. Cup anteversion was significantly lower in the DM group (11.1° vs. 14.1°; *p* = 0.006), while no significant differences were observed in LLD nor femoral offset between the groups (*p* = 0.38, *p* = 0.69, respectively). Dislocation rates were similar between the DM and conventional THA groups (1.2% vs. 1.02%, respectively; *p* = 0.54). Furthermore, revision rates were similar between DM and conventional THA (1.22% vs. 2.87%, respectively; *p* = 0.387). **Conclusions:** While no significant differences in dislocation rates were observed between dual-mobility and conventional THA bearings, the significantly lower cup anteversion suggests a potential improvement in acetabular safe zone positioning, this could reflect a broader margin for error in implant positioning. Further prospective studies are needed to elucidate the biomechanical advantages of DM bearings in patients with hip fractures.

## 1. Introduction

Total hip arthroplasty (THA) has become a widely accepted treatment for displaced femoral neck fractures, particularly in healthy and active patients, due to its ability to provide predictable pain relief, restore mobility, and improve patient-reported outcomes [1,2].

In recent years, the utilization of THA in the setting of femoral neck fractures has steadily increased, reflecting a shift in clinical practice patterns that favor THA over hemiarthroplasty for select patient populations; for example, recent epidemiological data indicate a marked rise in the proportion of femoral neck fracture patients treated with THA [3]. Unlike hemiarthroplasty, which preserves the native acetabulum, THA offers superior functional results and lower rates of acetabular erosion, making it an attractive option for physiologically robust older adults. However, despite these advantages, instability and dislocation remain among the most challenging and costly complications associated with THA, especially when performed in the trauma setting [4].

Dislocation rates following THA for hip fractures are substantially higher compared to those observed in elective procedures performed for osteoarthritis [5]. Some studies report rates up to five times greater in the fracture population, likely due to several factors unique to these patients, likely attributable to factors such as soft tissue injury sustained during the fracture, compromised periarticular structures, and the higher burden of comorbidities and frailty commonly present in this population [6]. Dislocation not only negatively affects individual patient outcomes—causing pain, loss of confidence in mobility, and increased dependence—but also imposes a significant burden on healthcare systems. These burdens stem from the need for revision surgeries, prolonged hospital stays, and extended rehabilitation periods, all of which contribute to increased morbidity and healthcare expenditures [7].

In an effort to mitigate the risk of postoperative instability, dual-mobility (DM) hip components have been introduced as an innovative implant design. These constructs incorporate an additional articulating surface, in which a polyethylene liner articulates within a metal shell, and the femoral head articulates within the liner itself [8]. This dual articulation increases the effective head-neck ratio, enhances the range of motion, and provides a greater “jump distance” before impingement or dislocation occurs. By combining these biomechanical advantages, DM implants are specifically designed to reduce the risk of instability compared with conventional fixed-bearing designs [9,10].

While DM components have demonstrated promising reductions in dislocation rates in elective primary and revision THA, their effectiveness in the setting of acute femoral neck fractures remains less well-established [11,12,13,14]. Evidence supporting their use in trauma patients has primarily been limited to retrospective cohort studies and smaller series, which may be subject to selection bias and lack sufficient power to detect meaningful differences. Additionally, hip fracture patients often present with complex clinical profiles, including advanced age, frailty, multiple comorbidities, cognitive impairment, and lower baseline functional status [15,16]. These factors complicate perioperative management, influence rehabilitation trajectories, and may differentially affect implant performance compared to healthier elective THA populations. Therefore, caution should be exercised when applying findings from elective THA populations to patients with hip fractures, as their distinct clinical characteristics may lead to different outcomes. Given the increasing adoption of THA as treatment for displaced hip fractures and the growing interest in dual-mobility technology as a strategy to improve stability, a direct comparison between DM bearings and conventional THA constructs in this context is warranted.

The present study aimed to compare the dislocation rates and early outcomes of dual-mobility bearings versus conventional THA components among patients undergoing primary THA for the treatment of displaced femoral neck fractures. In contrast to prior large-scale studies that primarily focused on clinical outcomes, the present study offers a detailed radiographic analysis—including cup anteversion, femoral offset, and leg length discrepancy—providing additional insight into implant positioning and its potential impact on stability.

## 2. Methods

### 2.1. Study Design

After receiving approval for this study from our Institutional Review Board, we retrospectively reviewed all patients who underwent THA for a displaced femoral neck fracture at a tertiary trauma center between January 2010 and December 2022. Between January 2022 and December 2022, our institutional protocol was modified and during that period, the preferred implants for THA in these patients included DM bearing implants. Only patients with a minimum postoperative follow-up of two years were included for analysis. Eligible patients were stratified into two groups by the type of bearing they received: (1) conventional bearing or (2) DM bearing.

### 2.2. Data Collection and Outcomes Measures

Patient baseline characteristics were systematically collected from our institution’s electronic medical record (EMR) database to ensure comprehensive and accurate data capture. The variables extracted included patients’ sex, chronological age at the time of surgery, body mass index (BMI) calculated in kilograms per square meter, Charlson Comorbidity Index (CCI) as an established measure of overall comorbidity burden, and the American Society of Anesthesiologists (ASA) physical status classification score to assess preoperative health status. These parameters were selected to facilitate an objective comparison between the study groups and to control for potential confounding factors that may influence outcomes.

**Figure 1:**
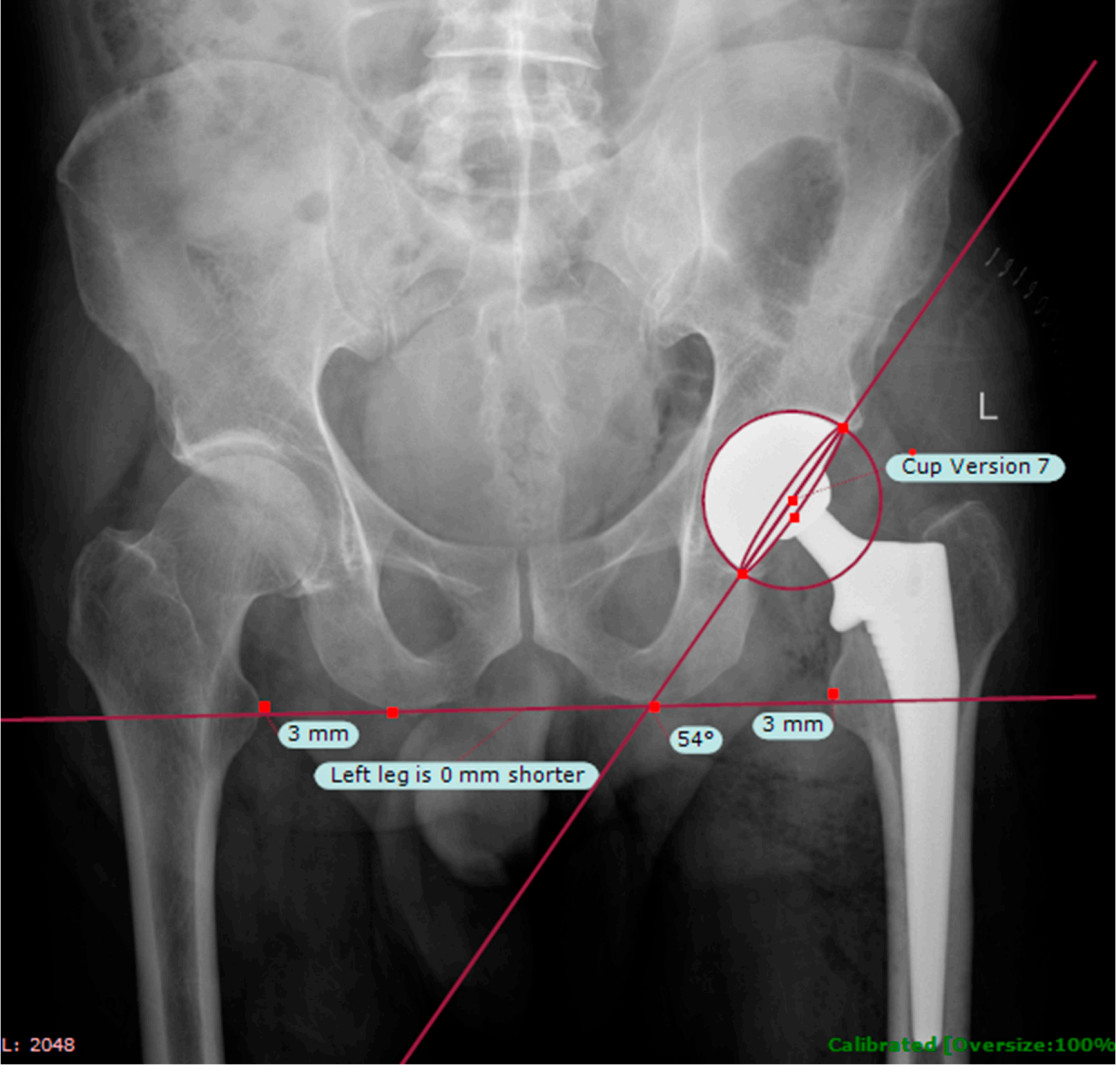
Cup anteversion and leg length discrepancy measurements using the postoperative TraumaCad^®^ assessment tool.

Postoperative radiographic assessments were performed to evaluate implant positioning and included specific measurements of cup anteversion and femoral horizontal offset, which are critical parameters in total hip arthroplasty biomechanics. Cup anteversion and leg length discrepancy (LLD) were measured using the TraumaCad^®^ software (version 2.0) platform, a validated digital templating tool that allows precise quantification of radiographic variables with minimal intraobserver and interobserver variability [17,18]. The consistent use of this software ensured reproducibility and accuracy of radiographic measurements across all patients in the study cohort (Figure 1).

**Figure 2:**
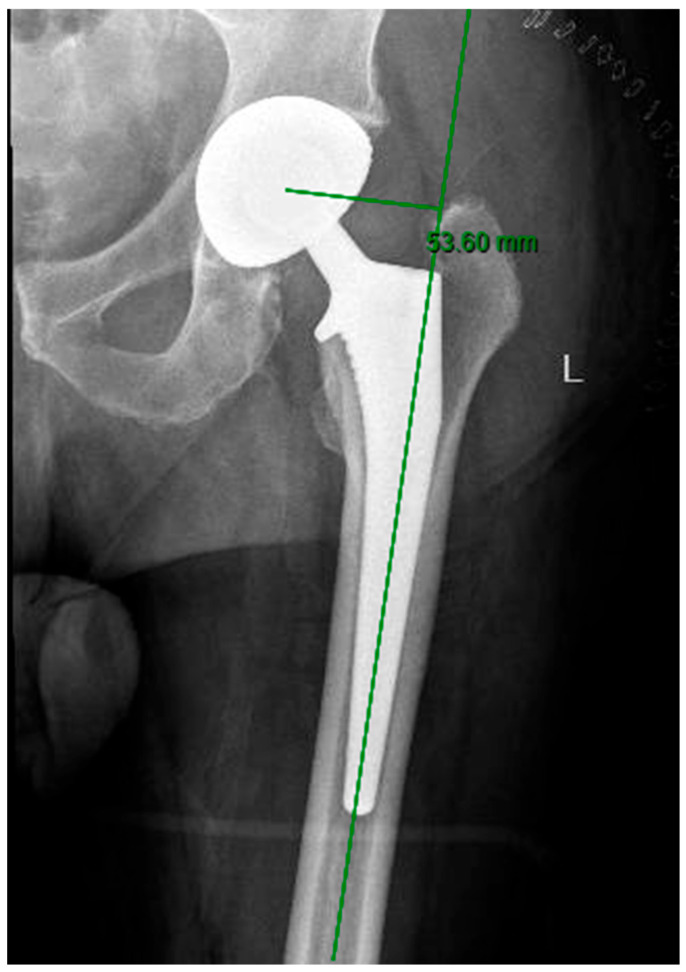
Femoral offset measurements using the postoperative TraumaCad^®^ assessment tool.

Femoral offset was determined by calculating the horizontal distance between the center of the femoral stem implant axis and the anatomical hip center of rotation. This measurement was obtained by drawing a line parallel to the reference line connecting the inferior-most points of the acetabular teardrops, thereby standardizing the orientation of the pelvic landmarks used for calibration (Figure 2) [19]. Accurate assessment of femoral offset is essential, as restoration of this parameter contributes to joint stability, soft tissue tension, and biomechanical efficiency following arthroplasty.

In addition to baseline and radiographic data, each patient’s EMR was carefully reviewed to determine the incidence of clinically significant postoperative events. Specifically, the rates of all-cause revision surgery and hip dislocations were recorded, as these constituted the primary outcomes of interest for this investigation. To minimize potential bias related to variable follow-up durations, these outcomes were consistently assessed at a minimum of two years postoperatively for all patients included in the final analysis cohort. This standardized follow-up interval ensured comparability of outcome data and enhanced the validity of the study findings. All procedures were performed by senior orthopedic surgeons with a minimum of five years of post-residency experience and formal fellowship training in arthroplasty. Patients who underwent dual mobility procedures received a BI-MENTUM Dual Mobility System implant (DePuy Synthes), whereas those who underwent conventional THA received a Pinnacle Sector II implant. All surgeries were performed through antero-lateral approach.

### 2.3. Statistical Analysis:

Continuous variables were summarized and reported as means with accompanying standard deviations to describe the central tendency and dispersion of the data. Categorical variables were expressed as absolute counts and corresponding percentages to illustrate the distribution of observations across categories. The normality of continuous variables was assessed using the Kolmogorov–Smirnov test, which evaluates whether the observed data significantly deviate from a normal distribution. Based on the outcome of this assessment, appropriate statistical tests were selected to compare the groups. Specifically, when the data were determined to follow a normal distribution, parametric testing was performed using the independent-samples t-test to detect differences in means between the two groups. For variables that did not meet the assumption of normality, the nonparametric Mann–Whitney U test was applied to evaluate differences in medians. For categorical variables, comparisons between groups were conducted using either the chi-squared test or Fisher’s exact test, as appropriate. The chi-squared test was utilized when the expected frequency counts in contingency tables were sufficient to meet the assumptions of the test, whereas Fisher’s exact test was employed in cases with small sample sizes or expected cell counts below five to ensure accuracy of *p*-value estimation. A two-tailed *p*-value of less than 0.05 was considered indicative of statistical significance for all analyses. All statistical analyses and data management were performed using Microsoft Excel (Microsoft Corporation, Redmond, WA, USA), which was used to organize datasets, execute the relevant statistical procedures, and generate descriptive and inferential statistics.

## 3. Results

A total of 642 patients who underwent primary total hip arthroplasty (THA) for a hip fracture were initially screened for inclusion. Following the exclusion of 72 patients due to insufficient follow-up duration, the final study population comprised 570 patients (89% of the initial cohort). Among these, 82 patients (14.4%) underwent THA with dual-mobility (DM) bearings, while 488 patients (85.6%) received conventional THA components.

Baseline demographic and clinical characteristics were similar between the two groups. There were no statistically significant differences in sex distribution (67% female in the DM group vs. 64.5% in the conventional group; *p* = 0.674), mean age (73.72 vs. 72.45 years; *p* = 0.211), mean body mass index (BMI) (26.40 vs. 25.33 kg/m^2^; *p* = 0.091), mean Charlson Comorbidity Index (CCI) (3.71 vs. 3.61; *p* = 0.648), or American Society of Anesthesiologists (ASA) class distribution (*p* = 0.567) (Table 1).

Radiographic measurements demonstrated that mean cup anteversion was significantly lower in the DM group compared to the conventional THA group (11.34° vs. 14.06°, respectively; *p* = 0.006). Mean leg length discrepancy (0.72 mm vs. 1.64 mm; *p* = 0.384) and mean femoral offset (44.92 mm vs. 45.31 mm; *p* = 0.697) were comparable between the groups.

Regarding clinical outcomes, there were no significant differences in the rates of dislocation within two years postoperatively (1.22% in the DM group vs. 1.02% in the conventional THA group; *p* = 0.873; 95% CI: 0.22–6.59). Similarly, revision rates at two years did not differ significantly between cohorts (1.22% for DM vs. 2.87% for conventional THA; *p* = 0.387).

## 4. Discussion

Dislocation following THA is a well-documented complication. While majority of studies have investigated this issue in elective patients, this study compared the outcomes of DM bearings to conventional THA in patients undergoing surgery for hip fractures. This study did not find a significant difference in dislocation rates or revision rates between the two groups.

Dislocation is a major complication following THA, particularly in the trauma population, where rates are substantially higher than in elective cases [20]. While elective THA dislocation rates range from 0.3 to 3%, trauma-related THA is associated with dislocation rates of 2–11% due to factors such as soft tissue compromise, poor bone quality, and higher rates of frailty and comorbidities [21,22]. In their propensity score matched study Jobory et al. reported on 4520 patients who were treated with DM for hip fractures, and reported on lower risk for revisions and dislocations when using a DM cup bearing [23,24,25]. Similarly, Cha et al. systematically reviewed 17 studies, including 2793 patients (2263 DM THA and 530 bipolar hip hemiarthroplasties for hip fractures) [26], showing a cumulative dislocation rate for DM THA at 4%. They concluded that DM-THA appeared to be a viable option for patients with displaced femoral neck fractures, with better reported rates of dislocation. Albanese et al. also conducted a systematic review, analyzing 7 comparative studies and concluding that the use of DM THA reduces the relative risk of dislocation by 83% when compared to conventional THA [14]. Contrary to previous studies, our findings show no statistically significant difference in dislocation rates between DM and conventional THA for hip fractures. This may be due to our relatively small cohort and lower dislocation rates compared to earlier research [26]. Further studies are needed to address these limitations.

Historically, implant positioning, particularly cup anteversion, have been critical in determining stability after THA [27,28]. Lewinnek et al. introduced the concept of a safe zone for implant positioning, defining an optimal range of acetabular cup placement that minimizes dislocation risk [29]. The current study’s findings show similar dislocation rates between groups, despite a significantly lower cup anteversion in the DM bearings group. This suggests a potential expansion of the safe zone range and improved implant stability with DM constructs. These findings raise important biomechanical considerations and suggest that dual mobility constructs may confer increased tolerance for malpositioning, potentially broadening the traditionally accepted safe zone. Ohmori et al. previously investigated this concept using a computed three-dimensional model, concluding that DM components could potentially expand the safe zone by 5–10 times [30]. Notably, the version values for both groups remained well within Lewinnek’s originally proposed safe zone (15° ± 10°), indicating that the observed version differences may lack clinical significance [29]. Moreover, contemporary research is expanding our understanding of the safe zone concept, exploring more nuanced approaches to evaluating implant positioning [31]. For instance, Tezuka et al. evaluated functional hip motion through lateral spine-pelvis-hip radiographs, highlighting the complexity of predicting hip joint stability, and that hip dislocation can occur even when cup angles appear within the traditionally defined “safe” parameters [32]. Therefore, DM constructs may offer enhanced stability in patients with dynamic pelvic motion or altered biomechanics—situations in which standard implants may be more vulnerable to dislocation. Taken together, these insights suggest that the lower anteversion observed in the DM group may not only be clinically acceptable but could reflect a broader margin for error in implant positioning. Further prospective, long-term studies are needed to definitively elucidate the potential biomechanical advantages of DM bearings in expanding the safe zone and improving hip stability in patients undergoing THA for a hip fracture.

This study has several limitations. Its retrospective nature may introduce biases related to data collection and patient selection. Lack of data on potential confounders such as surgeon experience, and detailed patient selection criteria, which may have influenced the outcomes and limited our ability to fully adjust for these factors in the analysis. Additionally, as a single-center study, the findings may not be fully generalizable to other institutions or settings with varying patient populations and surgical practices. Due to relatively modest cohort size and the low event rates, which may affect the robustness of certain statistical comparisons. Furthermore, the follow-up period, though sufficient for early complications, does not capture long-term outcomes such as implant wear or late dislocations. Moreover, surgeries were performed by multiple surgeons, which may introduce variability in surgical technique and outcomes and the study did not account for differences in implant size, which could impact the results. Lastly, even though the cohort size is big when compared to the current literature, it remains relatively small, limiting the generalizability of the findings. Further multi-center, prospective studies with longer follow-up are needed to validate these findings.

## 5. Conclusions

In conclusion, while the findings of this study demonstrated no significant differences in dislocation rates between dual-mobility and conventional THA bearings, significantly lower cup anteversion may improve the acetabular positioning safe-zone for the DM patient group. Further prospective, long-term studies are needed to definitively elucidate the potential biomechanical advantages of DM bearings in expanding the safe zone and improving hip stability in patients undergoing THA for a hip fracture.

Post-operative measurements were carried out using the TraumaCad^®^ assessment tool. Reference points for pelvic positioning were taken at the ischial tuberosities. For leg length measurements, landmarks were identified on the lesser trochanters (LT). Cup version was measured through points marked at the superolateral and inferomedial corners, as well as the most lateral point of the cup’s central portion.

Femoral offset measurement through an AP X-ray of the hip measuring the perpendicular distance from the center of the femoral head to the longitudinal axis of the femoral shaft.

## Figures and Tables

**Table 1:** Demographic, radiographic and clinical characteristics of DM vs. Standard THA for hip fractures.

	DM THA (*n* = 82)	Standard THA (*n* = 488)	*p*-Value
**Female Sex, *n* (%)**	55 (67)	315 (64.5)	0.674
**Mean age, years (SD)**	73.72 (8.39)	72.45 (8.98)	0.211
**Mean CCI (SD)**	3.71 (1.60)	3.61 (1.61)	0.648
**Mean BMI, Kg/m^2^ (SD)**	26.40 (5.15)	25.33 (4.27)	0.091
**ASA classification, *n* (%)**			0.567
1	1 (1.22)	33 (6.76)	
2	42 (51.22)	292 (59.84)	
3	35 (42.68)	152 (31.15)	
4	1 (1.22)	8 (1.64)	
**Mean LLD, mm (SD)**	0.72 (6.52)	1.64 (7.30)	0.384
**Mean Cup Anteversion, Deg (SD)**	11.34 (7.03)	14.06 (5.34)	**0.006**
**Mean Femoral Offset, mm (SD)**	44.92 (7.34)	45.31 (5.88)	0.697
**Dislocations, *n* (%)**	1 (1.22)	5 (1.02)	0.873
**Revision of any cause, *n* (%)**	1 (1.22)	14 (2.87)	0.387

DM, dual mobility; THA, total hip arthroplasty; CCI, Charlson Comorbidity Index; BMI, body mass index; ASA, American Society of Anesthesiologists; LLD, leg length discrepancy; Deg, degrees.

## Data Availability

The original contributions presented in this study are included in the article. Further inquiries can be directed to the corresponding author.
